# Supplementary material for: Relationship between the Dietary Inflammatory Index Score and Cytokine Levels in Chinese Pregnant Women during the Second and Third Trimesters
Source: Nutrients. 2022 Dec 30;15(1):194. doi: 10.3390/nu15010194 (PMC9824482; doi:10.3390/nu15010194)
Supplement: Supplementary file 1 [file nutrients-15-00194-s001.zip › nutrients-2025383-supplementary.pdf]

Supplementary Material

Table S1 Characteristics and DII scores of participants (n=175) *

	Participants (n=175)
**Gestational age at delivery (weeks)**	39.43(38.57,40.29)
**Sex of fetus**	
Male	70(40.00%)
Female	88(50.29%)
Missing	17(9.71%)
**Any complications**	
No	142(81.14%)
Yes	33(18.86%)
**Gravidity**	
1.00	115(65.71%)
2.00	43(24.57%)
3.00	17(9.71%)
**Parity**	
0.00	135(77.14%)
1.00	39(22.29%)
2.00	1(0.57%)

*Basic information of 175 pregnant women who participated in the cohort.

Table S2 Statistical analysis of restricted cubic spline regression between inflammatory parameters and DII scores in pregnant women in the second and third trimesters#

	T2	T3
CRP	0.792	0.696
IL-1β	0.148	**0.039***
IL-6	0.684	0.117
IL-8	0.104	0.306
IL-10	0.76	0.112
MCP-1	0.892	**0.035***
TNF-α	0.159	0.690

CRP, C-reactive protein; IL-1β, interleukin 1β; MCP-1, monocyte chemoattractant protein-1; T2, second trimester; T3, third trimester; TNF-α, tumor necrosis factor-α.

#*p* values for nonlinear dose–response relationship between DII score and various cytokines.

*$p < 0.05$

Table S3 Relationship between the change in repeated measurements of the DII and cytokines in pregnant women during the second and third trimesters of pregnancy

		**T3-T2**[#]	***p***
**CRP**	**P35-P65**	0.28±0.07	ref.
	**<P35**	0.43±0.08	0.172
	**≥P65**	0.45±0.07	0.098
**IL-10**	**P35-P65**	0.62±0.12	ref.
	**<P35**	0.52±0.12	0.557
	**≥P65**	**0.20*±0.11**	**0.011**
**IL-1β**	**P35-P65**	0.10±0.13	ref.
	**<P35**	-0.05±0.14	0.446
	**≥P65**	0.05±0.12	0.795
**IL-6**	**P35-P65**	0.16±0.08	ref.
	**<P35**	0.10±0.09	0.617
	**≥P65**	0.18±0.08	0.913
**IL-8**	**P35-P65**	0.51±0.08	ref.
	**<P35**	0.49±0.08	0.921
	**≥P65**	0.52±0.07	0.899
**MCP-1**	**P35-P65**	1.69±0.10	ref.
	**<P35**	1.77±0.11	0.620
	**≥P65**	1.77±0.09	0.564
**TNF-α**	**P35-P65**	0.64±0.11	ref.
	**<P35**	0.76±0.11	0.406
	**≥P65**	0.67±0.09	0.830

CRP, C-reactive protein; IL-1β, interleukin 1β; MCP-1, monocyte chemoattractant protein-1; T2, second trimester; T3, third trimester; TNF-α, serum tumor necrosis factor-α.

[#]T3-T2 for the mean DII difference between third and second trimester.

p value for the comparison between the <P35, ≥P65 and P35-P65 groups.

*$p < 0.017$

Table S4 The RNI and intake of major nutrients/food components and generalized linear analysis of nutrients/food components and DII scores

	T2				T3			
	**RNI^**	**Intake**	**%$**	**B(95%CI)#**	**RNI^**	**Intake**	**%$**	**B(95%CI)#**
β-carotenes(μg)	-	1140.76±1496.90	-	**0.00*(0.00,0.00)**	-	945.47±895.81	-	**0.00*(0.00,0.00)**
SFAs(g)	-	17.93±10.26	-	0.00(-0.02,0.02)	-	16.11±6.78	-	0.02(-0.02,0.07)
MUFAs(g)	-	18.50±11.90	-	0.00(-0.02,0.02)	-	15.31±8.73	-	-0.02(-0.06,0.02)
PUFAs(g)	-	13.09±12.96	-	0.00(-0.01,0.01)	-	8.62±6.47	-	0.01(-0.02,0.03)
Protein(g)	70.00	74.06±31.07	105.80	0.00(-0.01,0.01)	85.00	73.73±21.61	86.75	0.01(0.00,0.02)
Total fat(g)	-	48.18±25.96	-	**0.01*(0.00,0.02)**	-	46.62±20.53	-	0.01(0.00,0.02)
Carbohydrates(g)	-	231.76±98.79	-	0.00(0.00,0.00)	-	233.53±73.66	-	0.00(0.00,0.00)
Fiber (g)	-	11.97±6.94	-	**-0.05*(-0.07,-0.02)**	-	11.24±5.67	-	**-0.06*(-0.08,-0.03)**
Cholesterol(mg)	-	448.54±261.64	-	0.00(0.00,0.00)	-	465.76±224.11	-	0.00(0.00,0.00)
Vitamin A (RE)	770.00	1256.32±998.72	163.16	0.00(0.00,0.00)	770.00	1124.39±660.93	146.03	0.00(0.00,0.00)
Vitamin D (μg)	10.00	12.10±10.64	121.02	**-0.01*(-0.02,0.00)**	10.00	10.67±6.61	106.71	**-0.05*(-0.07,-0.03)**
Vitamin E (mg)	-	25.81±13.28	-	**-0.03*(-0.04,-0.02)**	-	24.95±10.60	-	**-0.03*(-0.05,-0.01)**
Thiamin(mg)	1.40	1.92±0.83	137.05	0.17(-0.20,0.54)	1.50	1.85±0.83	123.39	-0.40(-0.92,0.13)
Riboflavin(mg)	1.40	2.29±1.20	163.59	-0.03(-0.16,0.10)	1.50	2.15±1.02	143.50	**0.34*(0.06,0.62)**
Vitamin B6(mg)	2.20	1.89±1.23	85.82	**-1.31*(-1.99,-0.62)**	2.20	1.88±1.27	85.68	**-1.47*(-2.11,-0.83)**
Vitamin B12(μg)	2.90	2.63±1.90	90.81	**0.58*(0.15,1.00)**	2.90	2.60±1.93	89.80	**0.88*(0.41,1.36)**
Vitamin C(mg)	115.00	144.94±89.39	126.03	**0.00*(-0.01,0.00)**	115.00	139.54±92.72	121.33	0.00(0.00,0.00)
Folic acid(μg)	600.00	396.98±189.07	66.16	0.00(0.00,0.00)	600.00	333.99±233.61	55.67	**0.00*(0.00,0.00)**
Niacin(mg)	12.00	14.88±7.02	124.02	**-0.04*(-0.07,-0.01)**	12.00	14.14±5.60	117.81	0.00(-0.03,0.03)

Mg(mg)	370.00	344.45±130.23	93.10	**0.00*(-0.01,0.00)**	370.00	338.06±110.12	91.37	**0.00*(-0.01,0.00)**
Fe(mg)	24.00	51.29±30.56	213.70	**-0.01*(-0.02,0.00)**	29.00	51.11±30.75	176.23	0.00(-0.01,0.01)
Zn(mg)	9.50	13.50±5.66	142.12	**0.07*(0.03,0.11)**	9.50	13.83±4.92	145.58	-0.03(-0.10,0.03)
Se(μg)	65.00	54.02±28.27	83.11	0.00(-0.01,0.00)	65.00	54.14±19.64	83.29	**-0.01*(-0.02,0.00)**

CRP, C-reactive protein; Fe, iron; IL-1β, interleukin 1β; MCP-1, monocyte chemoattractant protein-1; Mg, magnesium ; MUFAs, monounsaturated fatty acids; PUFAs, polyunsaturated fatty acids; Se, selenium ;SFAs, saturated fatty acids; T2, second trimester; T3, third trimester; TNF-α, tumor necrosis factor-α; Zn, zinc.

^ RNI for WS/T 578.1—2017, Chinese dietary reference intake—Part 1: Macronutrients; WS/T 578.2—2018, Chinese dietary reference intake—Part 2: Macroelements; WS/T 578.3—2017, Chinese dietary reference intake—Part 3: Trace elements; WS/T 578.4—2018, Chinese dietary reference intake—Part 4: Lipid-soluble vitamins; WS/T 578.5—2018, Chinese dietary reference intake—Part 5: Water-soluble vitamins

[$] % for intake divided by RNI and multiplied by 100

[#] Coefficient generalized linear model analysis of the effects of major nutrients/food ingredients on DII.

* $p < 0.05$
